# Improvement of Pitting-Corrosion Resistance of Ultrafine-Grained 7475 Al Alloy by Aging

**DOI:** 10.3390/ma15010360

**Published:** 2022-01-04

**Authors:** Ewa Ura-Bińczyk

**Affiliations:** Faculty of Materials Science and Technology, Warsaw University of Technology, ul. Wołoska 141, 02-747 Warsaw, Poland; ewa.ura@pw.edu.pl

**Keywords:** 7475 aluminium alloy, pitting corrosion, SPD, grain refinement

## Abstract

The effect of aging on the resistance to pitting corrosion of ultrafine-grained 7475 aluminium (Al) alloy processed by hydrostatic extrusion (HE) is studied. Differences in the microstructure were investigated using secondary electron (SEM) and transmission electron microscopy (TEM). Corrosion tests were performed in 0.1 M NaCl, and characterization of corroded surface was performed. The results of this work show that the pitting susceptibility of ultra-fine grained 7475Al is related to the distribution of MgZn_2_ precipitates. After HE, the formation of An ultrafine-grained microstructure at the grain boundaries of ultrafine grains is observed, while subsequent aging results in the formation of MgZn_2_ precipitates in the grain interior. Grain refinement increases susceptibility to localized attack, while the subsequent aging improves the overall corrosion resistance and limits the propagation of corrosion attack.

## 1. Introduction

Severe plastic deformation (SPD) techniques by the creation of nano- and ultrafine-grained, nonporous, metallic materials are methods of grain-size refinement leading to improvement of their mechanical properties [1,2] The most common SPD methods are equal-channel angular pressing (ECAP) [3] and high-pressure torsion (HPT) [4]. Other methods, such as calibre rolling [5] or hydrostatic extrusion (HE) [6] are also efficient in terms of microstructure refinement and enhancement of mechanical properties. The main advantage of HE is the ability to produce nano- and ultrafine-grained bulk products of brittle or hard-to-deform materials [7], such as age hardenable 7000 series Al alloys. Their high mechanical strength is a result of precipitation hardening occurring during aging [8], and their maximum strength can be obtained by artificial aging to peak strength (T6 temper) [9]. The 7475 Al alloy owes its attractiveness to high strength, fracture toughness, and fatigue-crack-propagation resistance in air and destructive environments. Nevertheless, due to its chemical composition (Al-Zn-Mg-Cu), 7475 is prone to corrosion [10]. Therefore, new possibilities concerning improvement of its corrosion resistance by optimal selection of SPD parameters and post-processing variables are still being investigated. The effect of grain refinement resulting from SPD processing on the corrosion resistance for different Al alloys is still unclear. Generally, grain-size reduction results in a significant increase in volume fraction of the intracrystalline regions, such as grain boundaries and triple junctions [11,12], which are more chemically active than grain interiors [13,14,15,16]. This leads to the accelerated formation of passive films [17]. However, under conditions where the material undergoes active dissolution, a higher share of intercrystalline regions may enhance the corrosion rate of Al alloys [18,19]. SPD methods also lead to fragmentation and a more uniform distribution of intermetallic particles in Al alloys with heterogeneous microstructures [20,21,22]. The galvanic coupling of intermetallic particles with the surrounding matrix leads to the initiation of pitting [23,24]. The fragmentation of intermetallic particles is often related to the improvement of corrosion resistance of Al alloys due to the formation of a more continuous passive film and reduction in micro-galvanic currents [25,26,27,28]. However, it has also been reported that the multiplication of pit initiation sites led to decreased resistance to pitting corrosion of ECAP-processed Al alloys [29,30,31]. The other microstructural features influencing corrosion behaviour of Al alloys are related to the different processing conditions, whereby various factors may change the corrosion susceptibility of Al alloys in a significant way. As shown by Nickel et al. [32], corrosion attack can be affected by strain localization accumulated during single-step ECAP processing. Similar observations were made by Ly et al. [33] for the 6061 Al alloy after seven ECAP passes. A statistical analysis of the pitting susceptibility of the ultrafine-grained 1050 Al alloy according to the number of ECAP passes was performed by Quartiermeister et al. [34]. The authors stated that pitting-corrosion resistance was improved after eight ECAP passes. Aging heat treatment has a crucial impact on the corrosion susceptibility of 7000 series Al alloys. Al-Zn-Mg-Cu alloy usually obtains the highest strength after peak-aged T6 heat treatment. However, this heat treatment leads to severe intergranular corrosion (IGC) due to stable η-MgZn_2_ precipitates formed continuously along grain boundaries [35,36]. SPD processing alters the precipitation mechanism in age-hardenable alloys [37,38,39,40]. For cryo-rolled Al-4Zn-2Mg alloy, a significant improvement in resistance to IGC was reported [41].

The necessity of increased strength and corrosion resistance of Al alloy leads to the search for ever-new solutions to control the microstructure of alloys by selection of optimal post-processing techniques. It was revealed that further enhancement of mechanical strength can be obtained by combining grain refinement with subsequent aging. However, the effect of aging on resistance to localized attack of ultrafine-grained 7000 series Al alloys is not yet defined. Therefore, the present study reveals the effect of aging on the corrosion resistance of ultrafine-grained 7475 Al alloy. The influence of various microstructural factors on pitting susceptibility of 7475 Al alloy is investigated in terms of various post-processing routes. Three variously processed materials were examined: (1) coarse-grained, precipitation-strengthened, (2) ultrafine-grained directly after HE, and (3) ultrafine-grained, precipitation-strengthened (aged after HE).

## 2. Materials and Methods

### 2.1. Materials

The material used in this study was commercially available 7475 Al alloy (Al-Zn-Mg-Cu) with the chemical composition given in Table 1.

The material was delivered in the form of extruded rods. First, the material was annealed to homogenize the microstructure. The billets cut from these rods were subjected to HE in a multi-step process. The hydrostatic extrusion process was carried out at the Institute of High Pressure Physics, Polish Academy of Sciences, Warsaw, Poland.

In this study, three types of samples were investigated, namely:

- CG_T6—coarse-grained, precipitation-strengthened: coarse-grained sample after solution annealing at 470 °C for 2 h, water quenching, and peak aging;
- HE—ultrafine-grained, naturally aged: ultrafine-grained sample after solution annealing at 470 °C for 2 h, water quenching, and hydrostatic extrusion in three consecutive passes from the initial diameter of 20 mm to the final diameter of 3 mm, which corresponds to a total true strain of about 4;
- HT—ultrafine-grained, artificially aged: the same sample as described in point B, additionally aged at 100 °C for 54 h, which corresponds to the peak aging [37,43].

The microhardness of the materials after proposed post-processing was found to be HV_0.2_ = 180 for the CG_T6 alloy, HV_0.2_ = 180 for the HE material, and HV_0.2_ = 198 for the HT alloy [42]. The microhardness values indicate that the post-processing method of the alloy was properly chosen, and during additional annealing precipitation, strengthening took place. 

The microscopic observations and electrochemical measurements were performed on cross-sections of the rods, perpendicular to the HE direction. 

### 2.2. Microstructural Observations

A transmission electron microscope (TEM, JOEL, JEM-1200EX, Tokyo, Japan) operating at 120 kV was utilized to observe the refined microstructures of the alloys. To approach this, the thin films were prepared using a Gatan Model 656 Dimple Grinder and Gatan Model 691 Precision Ion Polishing System (PIPS, Ametek GmbH, Unterschleissheim, Germany). 

The microstructural observations of CG-T6 sample and post-corrosion morphology were performed using a field emission scanning electron microscope (FE-SEM, Hitachi SU-70, Hitachi, Tokyo, Japan). An energy-dispersive spectrometer (EDS) was used to perform chemical analyses of intermetallic phases. 

### 2.3. Electrochemical Testing

Electrochemical measurements were performed in a naturally aerated quiescent 0.1 M NaCl solution using Autolab PGSTAT302N potentiostat/galvanostat (Metrohm, Herisau, Switzerland). A standard three-electrode setup with a platinum sheet as a counter electrode (CE), a silver chloride electrode (Ag|AgCl|Cl^−^) as a reference electrode (RE), and a sample as a working electrode (WE) was used. An open-circuit potential (*E_OCP_*) was recorded during 45 h of immersion in the solution. Potentiodynamic polarization was carried out after 30 min of immersion, started at 0.25 V below *E_OCP_* with a 1 mV/s scan rate, and stopped when the potential reached a value of 0 V/Ref. Tafel extrapolation was used to obtain the characteristic parameters of the polarization curve (±10 mV around E_corr_). Prior to microscopic observations and electrochemical testing, the samples were ground to 2500# SiC papers and then polished with a diamond suspension from 3 to 1 µm with water-free lubricant. Finally, samples were ultrasonically cleaned in ethanol. Each measurement was repeated at least 3 times to ensure the reproducibility of the results. 

## 3. Results

### 3.1. Microstructure Characterization

#### 3.1.1. Grain Size and Strengthening Precipitates

**Figure 1:**
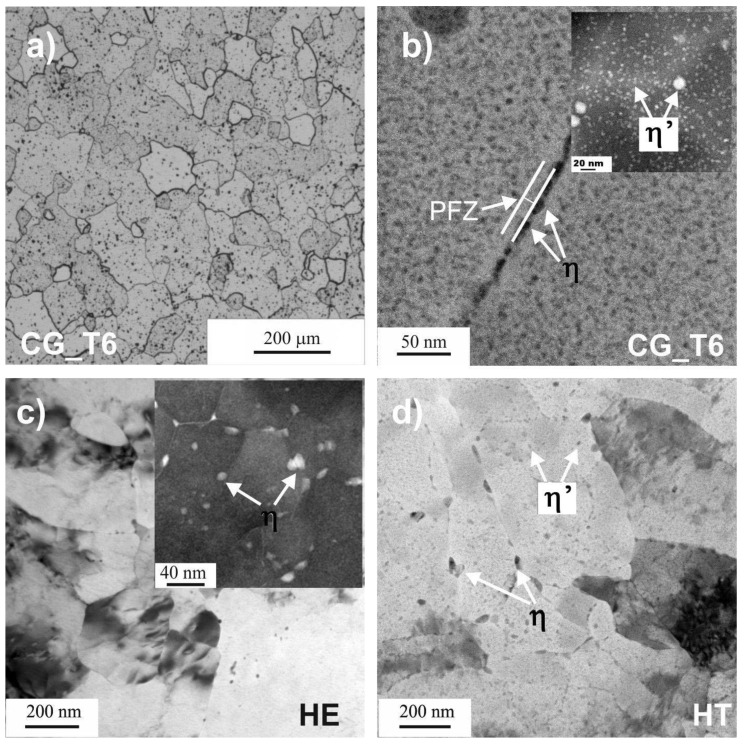
Microstructure of the 7475 Al alloy: (**a**) SEM microstructure of the CG_T6 sample, (**b**) TEM microstructure of the CG_T6 sample, (**c**) TEM microstructure of the HE sample, (**d**) TEM microstructure of the HT sample.

The microstructure of the CG_T6 sample consisted of equiaxed grains with an average diameter of about 40 µm (Figure 1a). As is usual in the 7000 Al series, η-MgZn_2_ precipitates were located at grain boundaries, surrounded by the precipitation-free zone (PFZ) [35]. Additionally, fine η’-MgZn_2_ precipitates were homogeneously distributed in grain interiors (insert in Figure 1b) [37,43]. 

HE resulted in a significant grain refinement, with high inhomogeneity in grain size (Figure 1c). Regions with well-developed grains with a diameter of about 70 nm and the areas with a less advanced stage of grain refinement (larger grain of about 1 µm in diameter with a relatively high density of dislocations inside) were observed. After natural aging, the precipitates of η-MgZn_2_ were observed at grain boundaries and triple points of well-developed grains (shown in the inset in Figure 1c) [37]. The grain interiors were free of precipitates. After artificial aging (Figure 1d), small precipitates in the grain interiors and extensive precipitation at grain boundaries were noticeable. At grain boundaries, the η-MgZn_2_ phase was precipitated, whilst in the grain interiors, η’-MgZn_2_ was created [37]. The precipitates formed at the grain boundaries were noticeably larger than those observed in the grain interiors.

#### 3.1.2. Intermetallic Particles

**Figure 2:**
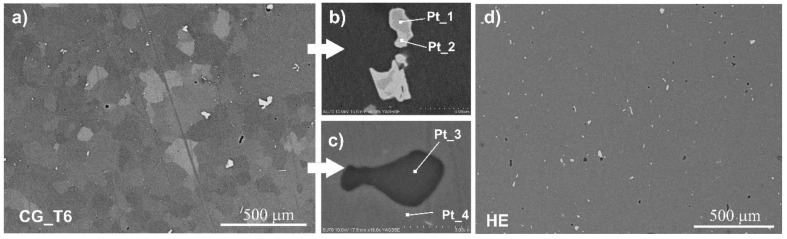
SEM images of intermetallic particles in the 7475 Al alloy: (**a**) SEM image of the CG_T6 sample, (**b**) BSE image of the Al-Cu-Fe particle, (**c**) BSE image of the Mg_2_Si particle, (**d**) SEM image of the HE sample.

As shown in Figure 2, two types of coarse intermetallic particles were found: bright Al-Cu-Fe particles (Figure 2b) and dark Mg_2_Si particles (Figure 2c). Their chemical composition is given in Table 2. In the coarse-grained sample (Figure 2a), Al-Cu-Fe particles had an average diameter of 1.4 µm and exhibited a great diversity in size, as quantified by the coefficient of variation, C_V_ (the ratio of the standard deviation to the mean value), which was 0.6. The exemplary image of intermetallic particles of Mg_2_Si is shown in Figure 2c. 

Intermetallic particles were distributed uniformly in all samples, as illustrated in Figure 2a,d. Our previous studies revealed that after HE, the average size of Al-Cu-Fe particles decreased to 1.2 µm, and their size distribution became narrower (the C_V_ parameter was reduced to 0.3) [43]. Intermetallic particles are not affected by aging [44].

### 3.2. Corrosion Resistance

#### 3.2.1. Corrosion under Open-Circuit Conditions

**Figure 3:**
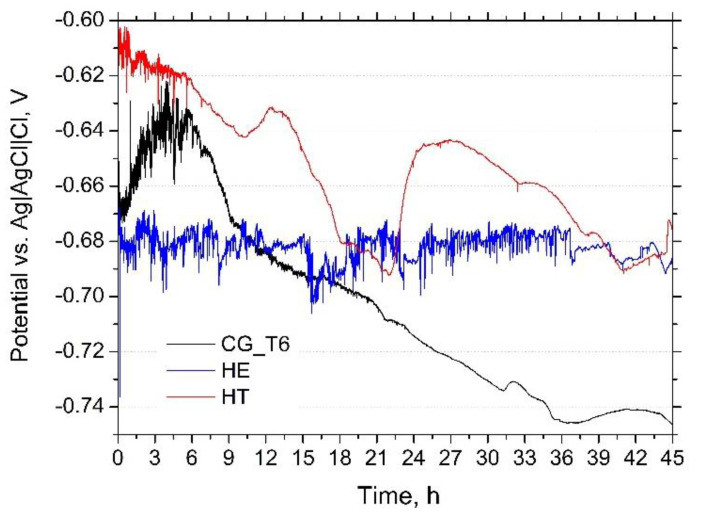
*E_OCP_* vs. time recorded during 45 h immersion of CG_T6, HE and HT 7475 Al alloy in 0.1 M NaCl.

The changes of *E_OCP_* recorded during 45 h immersion in naturally aerated 0.1 M NaCl are shown in Figure 3. For the CG_T6 sample, *E_OCP_* increased during the initial 5 h, and the oscillations of *E_OCP_* were noticeable. After 5 h, the potential had gradually decreased and finally achieved a value of about −0.75 V/Ref. For the sample after HE, *E_OCP_* was stable over the entire time of the immersion and was oscillating around −0.67 V/Ref. The oscillations were about ±0.05 V/Ref, higher than those observed for the CG_T6 sample. These observations are in contradiction to data obtained for pure Al [25,45], where the fluctuations of *E_OCP_* were attenuated for the fine-grained Al and related to the grain-size-reduction-induced transition from pitting to uniform corrosion. The subsequent aging shifted the *E_OCP_* of the HT sample to more noble values; however, during the entire immersion, a stable decrease in *E_OCP_* was indicated. 

#### 3.2.2. Potentiodynamic Polarization 

**Figure 4:**
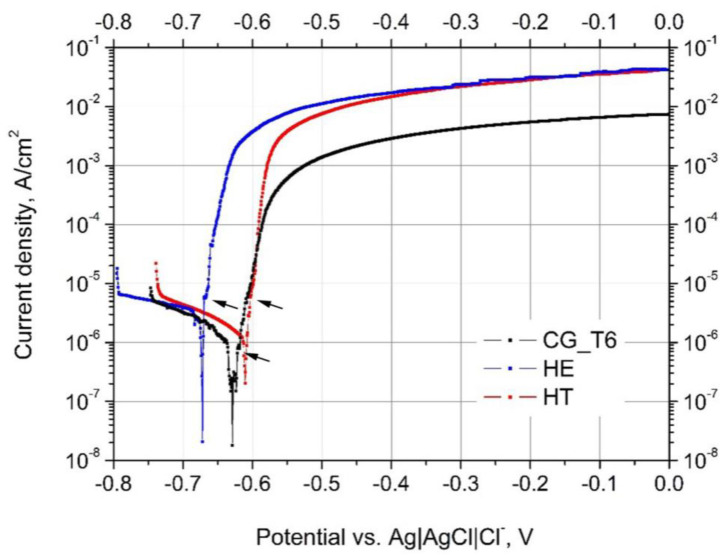
Potentiodynamic polarization curves of the 7475 Al alloy in an aerated 0.1 M NaCl.

The potentiodynamic polarization curves, recorded after 0.5 h stabilization at *E_OCP_* for CG_T6, HE, and HT samples, are plotted in Figure 4. The cathodic ranges of potentiodynamic curves recorded for CG_T6 and HE alloys were overlapped at the same current values, indicating that cathodic reactions were similar. A slight shift of the cathodic part of the curve towards higher current values was observed for the HT 7475 Al alloy. The shape of the anodic curves was similar for all the samples: a rapid increase in current density indicates that 7475 Al alloy was prone to localized attack and pitting occurred immediately after *E_corr_* was reached. Table 3 shows the corrosion potential (*E_corr_*) and pitting potentials (*E_pit_*) for all of the samples. *E_pit_* is marked with arrows in Figure 4. Neither of the alloys showed the typical passivation behaviour; however, even a slight difference between *E_corr_* and *E_pit_* can give a specific information about pitting susceptibility. In the case of the tested materials, the differences were small, with the highest value of 6 mV calculated for the HT 7475 Al alloy, suggesting that in all polarization tests, the alloys pitted as soon as they were placed into solution, showing a very short passive region and hence a very narrow safety margin. Nevertheless, the greater the difference between free and pitting potentials, the higher the resistance to pitting corrosion [46,47,48]. These findings indicate that additional aging limits the pitting tendency of the HT 7475 Al alloy. 

### 3.3. Post-Corrosion Morphology

**Figure 5:**
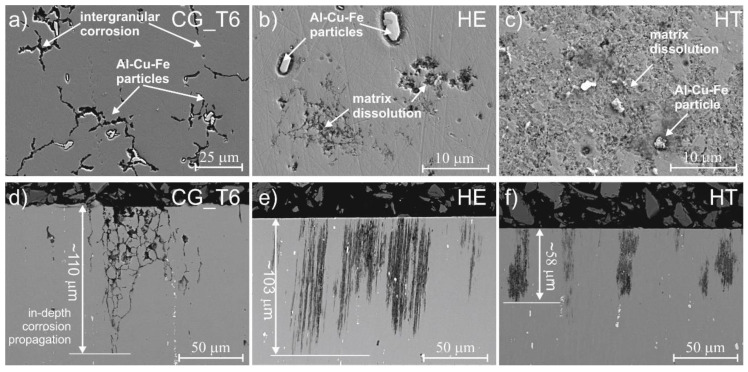
Post-corrosion morphology on the surface (**a**–**c**) and on the cross sections (**d**–**f**) of CG_T6, HE, and HT 7475 Al alloys after potentiodynamic polarization in aerated 0.1M NaCl.

The morphology of the corrosion attack after potentiodynamic polarization is shown in Figure 5. Dissolution of the matrix adjacent to the Al-Cu-Fe particles and IGC was observed on the surface of the CG_T6 sample. Due to the presence of copper, the Al-Cu-Fe intermetallic particles are cathodic, leading to galvanic coupling with the anodic matrix. The corrosion attack propagated around 110 μm in depth of the material, and this was typical IGC along the grain boundaries (Figure 5d). Similar peripherical corrosion around Al-Cu-Fe particles occurred on the surface of the HE sample (Figure 5b). Additionally, a dissolution of the matrix in random areas, where no large Cu- and Fe-rich particles were observed, also occurred. The corrosion depth was similar to that of the CG_T6 sample (Figure 5e), but the morphology of the corrosion attack was different. The corrosion propagation paths appeared as thin, straight lines arranged in bands, and they were formed along the direction of HE. A corrosion attack around the intermetallic particles was also observed on the surface of the HT sample (Figure 5c). Compared to the other samples, the main difference was that a large area of the matrix also dissolved, which indicates that the corrosion attack spread more in the latter. Cross-sectional observations show that in-depth corrosion propagation was shallower than for HE and CG_T6 samples (Figure 5f). The corrosion attack also propagated along the extrusion direction, but it appeared that the dissolution of the material spread more in the latter than in depth.

## 4. Discussion

In this work, the pitting susceptibility of three 7475 Al alloys was investigated. In the first alloy with coarse grains (CG_T6), precipitation of η-MgZn_2_ at grain boundaries surrounded by PFZs, and and η’-MgZn_2_ was mainly observed in the grain interiors. The second alloy was processed via HE, characterized by UFG microstructure and the presence of η-MgZn_2_, while the third alloy, marked as HT, was additionally aged with both η-MgZn_2_ and η’-MgZn_2_ precipitates. Al-Cu-Fe and Mg_2_Si particles existed in all tested materials. Regardless of the grain size, the corrosion attack observed on all the samples occurred at the interface between Cu- and Fe-rich particles and a matrix, leading to the dissolution of the matrix. The presence of a stable MgZn_2_ phase (η) along the grain boundaries and PFZ resulted in a localized attack propagating along grain boundaries [36,49,50,51]. For the deformed material (HE), microstructure refinement changed the distribution of the strengthening precipitates. In the 7475 Al alloy after HE, only nanoscale MgZn_2_ precipitates at the grain boundaries were present, but their density was relatively small compared to the reference CG_T6 alloy. The most negative *E_OCP_* recorded for the HE sample might be related to a higher concentration of Mg and Zn in the matrix, which tends to lower *E_OCP_* of Al alloys [52]. The subsequent aging leads to the formation of an intragranular metastable η’ phase, the presence of which shifts *E_OCP_* towards more positive values than for the HE and CG_T6 samples and lowers the corrosion current. *E_OCP_* of the refined material without subsequent aging was stable upon long exposure, while for the HT sample, it gradually decreased during immersion, similarly to the CG_T6 sample. 

### Pitting Susceptibility

The obvious differences in the post-corrosion observations revealed that corrosion of the alloys was strongly related to the utilized post-processing method. For the reference CG_T6 material, the corrosion attack propagated towards the depth of the material and along grain boundaries. In the HE 7475 Al and HT 7475 Al alloys, the corrosion attack propagated along the extrusion direction and was arranged in bundles of thin strings perpendicular to the surface. Generally, the typical microstructure of the extruded materials is composed of the elongated grains towards the extrusion direction, which resembles a fibrous grain structure [53]. The subsequent aging limited the corrosion propagation towards the bulk material, which may be related to the continuity of grain boundaries along the extrusion direction that might be disrupted due to coarsening of the stable η phase and thus stopping further dissolution along the grains. Therefore, material dissolved more in the latter condition.

## 5. Conclusions

Based on the results of this work, the following conclusions can be drawn:

- The HE7475 Al alloy developed an ultrafine-grained structure with inhomogeneous grain size, coarse intermetallic particles, and MgZn_2_ strengthening precipitates at grain boundaries.
- Subsequent aging resulted in coarsening of stable intergranular MgZn_2_ precipitates and formation of metastable MgZn_2_ in grain interiors. The lower number of strengthening particles shifted *E*_OCP_ to less noble values as a higher amount of Mg and Zn was dissolved in the matrix. The aging improved the corrosion resistance as the *E*_OCP_ was shifted to more noble values and the *i_corr_* was lower.
- The resulting HE microstructural changes significantly altered the morphology of the corrosion attack. The IGC was no longer observed for ultrafine-grained materials. The subsequent aging limited the propagation of the corrosion attack towards the depth of the bulk material.
- The subsequent aging seems to have had an impact on the pitting susceptibility of the ultrafine-grained 7475 Al alloy.

## Figures and Tables

**Table 1:** Chemical composition of 7475 Al alloy (wt.%) [42].

Alloy	Zn	Mg	Cu	Zr	Fe	Si	Ti	Mn
7475	6.00	2.49	1.66	0.12	0.12	0.094	0.015	0.01

**Table 2:** EDS analysis of the chemical composition of secondary phases present in the 7475 Al alloy (points 1–4 in Figure 3).

% at.	Mg-K	Al-K	Si-K	Fe-L	Cu-L	Zn-L
Pt_1	0.4 ± 0.1	66.4 ± 0.2	2.5 ± 0.1	22.2 ± 0.1	8.5 ± 0.1	-
Pt_2	-	61.0 ± 0.2	-	15.0 ± 0.2	24.0 ± 0.2	-
Pt_3	60.2 ± 0.3	2.1 ± 0.1	37.8 ± 0.3	-	-	-
Pt_4	2.8 ± 0.1	92.5 ± 0.4	-	0.2 ± 0.1	0.7 ± 0.1	3.8 ± 0.1

**Table 3:** Average electrochemical parameters: corrosion potential (*E_corr_*), pitting potential (*E_pit_*), and corrosion current density *(i_corr_*) obtained from potentiodynamic curves.

Sample	*E_corr_* vs. Ag|AgCl|Cl^−^, V	*E_pit_* vs. Ag|AgCl|Cl^−^, V	*i_corr_*, µA/cm^2^
CG_T6	−0.629 ± 0.02	−0.626 ± 0.2	1.0 ± 0.2
HE	−0.672 ± 0.01	−0.669 ± 0.1	3.4 ± 0.2
HT	−0.610 ± 0.01	−0.604 ± 0.1	1.4 ± 0.1

## Data Availability

Not applicable.

## References

[1] Valiev R.Z., Islamgaliev R.K., Alexandrov I.V. (2000). Bulk nanostructured materials from severe plastic deformation. Prog. Mater. Sci..

[2] Hansen N. (2004). Hall-petch relation and boundary strengthening. Scr. Mater..

[3] Valiev R.Z., Langdon T.G. (2006). Principles of equal-channel angular pressing as a processing tool for grain refinement. Prog. Mater. Sci..

[4] Zhilyaev A.P., Langdon T.G. (2008). Using high-pressure torsion for metal processing: Fundamentals and applications. Prog. Mater. Sci..

[5] Lee J.H., Kwak B.J., Kong T., Park S.H., Lee T. (2019). Improved tensile properties of AZ31 Mg alloy subjected to various caliber-rolling strains. J. Magnes. Alloy..

[6] Lewandowska M., Kurzydlowski K.J. (2008). Recent development in grain refinement by hydrostatic extrusion. J. Mater. Sci..

[7] Lewandowska M. (2006). Mechanism of Grain Refinement in Aluminium in the Process of Hydrostatic Extrusion. Solid State Phenom..

[8] MacKenzie D.S., Anderson K., Weritz J., Kaufman J.G., Mackenzie D.S. (2018). Metallurgy of heat treatable aluminum alloys. ASM Handbook Volume 4E: Heat Treating of Nonferrous Alloys.

[9] Totten G.E. (2016). Heat Treatment Practice of Age-Hardenable Aluminium Alloys. ASM Handbook Volume 4E: Heat Treating of Nonferrous Alloys.

[10] Emmanuel A.O., Fayomi O.S.I., Akande I.G. (2021). Aluminium Alloys as Advanced Materials: A short communication. IOP Conf. Ser. Mater. Sci. Eng..

[11] Palumbo G., Aust K.T., Erb U. (1996). Triple line defects in nanostructured materials. Mater. Sci. Forum..

[12] Palumbo G., Thorpe S.J., Aust K.T. (1990). On the contribution of triple junctions to the structure and properties of nanocrystalline materials. Scr. Metall. Mater..

[13] Liao J., Hotta M., Yamamoto N. (2012). Corrosion behavior of fine-grained AZ31B magnesium alloy. Corros. Sci..

[14] Abdulstaar M., Mhaede M., Wagner L., Wollmann M. (2014). Corrosion behaviour of Al 1050 severely deformed by rotary swaging. Mater. Des..

[15] Wang S.-S., Yang F., Frankel G.S. (2017). Effect of Altered Surface Layer on Localized Corrosion of Aluminum Alloy 2024. J. Electrochem. Soc..

[16] Dobkowska A., Castillo M.D.H., Turnbull J.P., Ramamurthy S., Zagidulin D., Moser D.E., Behazin M., Keech P.G., Shoesmith D.W., Noël J.J. (2021). A comparison of the corrosion behaviour of copper materials in dilute nitric acid. Corros. Sci..

[17] Ralston K.D., Fabijanic D., Birbilis N. (2011). Effect of grain size on corrosion of high purity aluminium. Electrochim. Acta.

[18] Ralston K.D., Birbilis N., Davies C.H.J. (2010). Revealing the relationship between grain size and corrosion rate of metals. Scr. Mater..

[19] Orlov D., Ralston K.D., Birbilis N., Estrin Y. (2011). Enhanced corrosion resistance of Mg alloy ZK60 after processing by integrated extrusion and equal channel angular pressing. Acta Mater..

[20] Adamczyk-Cieślak B., Mizera J., Kurzydłowski K.J. (2011). Microstructures in the 6060 aluminium alloy after various severe plastic deformation treatments. Mater. Charact..

[21] Crump J., Qiao X.G., Starink M.J. (2012). The effect of high-pressure torsion on the behaviour of intermetallic particles present in Al-1Mg and Al-3Mg. J. Mater. Sci..

[22] Shaterani P., Zarei-Hanzaki A., Fatemi-Varzaneh S.M., Hassas-Irani S.B. (2014). The second phase particles and mechanical properties of 2124 aluminum alloy processed by accumulative back extrusion. Mater. Des..

[23] Hughes A.E., Birbilis N., Mol J.M.C., Garcia S.J., Zhou X., Thompson G.E. (2011). High Strength Al-Alloys: Microstructure, Corrosion and Principles of Protection. Recent Trends in Processing and Degradation of Aluminium Alloys.

[24] Birbilis N., Buchheit R.G. (2005). Electrochemical Characteristics of Intermetallic Phases in Aluminum Alloys. J. Electrochem. Soc..

[25] Jilani O., Njah N., Ponthiaux P. (2014). Transition from intergranular to pitting corrosion in fine grained aluminum processed by equal channel angular pressing. Corros. Sci..

[26] Sikora E., Wei X.J., Shaw B.A. (2004). Corrosion behavior of nanocrystalline bulk Al-Mg-based alloys. Corrosion.

[27] Chung M.K., Choi Y.S., Kim J.G., Kim Y.M., Lee J.C. (2004). Effect of the number of ECAP pass time on the electrochemical properties of 1050 Al alloys. Mater. Sci. Eng. A.

[28] Akiyama E., Zhang Z., Watanabe Y., Tsuzaki K. (2009). Effects of severe plastic deformation on the corrosion behavior of aluminum alloys. J. Solid State Electrochem..

[29] Korchef A., Kahoul A. (2013). Corrosion Behavior of Commercial Aluminum Alloy Processed by Equal Channel Angular Pressing. Int. J. Corros..

[30] Laurino A., Andrieu E., Harouard J.-P., Lacaze J., Lafont M.-C., Odemer G., Blanc C. (2013). Corrosion Behavior of 6101 Aluminum Alloy Strands for Automotive Wires. J. Electrochem. Soc..

[31] Pisarek M., Kędzierzawski P., Janik-Czachor M., Kurzydłowski K.J. (2009). Effect of hydrostatic extrusion on passivity breakdown on 303 austenitic stainless steel in chloride solution. J. Solid State Electrochem..

[32] Nickel D., Dietrich D., Mehner T., Frint P., Spieler D., Lampke T. (2015). Effect of strain localization on pitting corrosion of an AlMgSi0.5 alloy. Metals.

[33] Ly R., Hartwig K.T., Castaneda H. (2018). Influence of dynamic recrystallization and shear banding on the localized corrosion of severely deformed Al–Mg–Si alloy. Materialia.

[34] Quartiermeister M.V., Magalhães D.C.C., Vacchi G.S., Braga D.P., Silva R., Kliauga A.M., Sordi V.L., Rovere C.A.D. (2020). On the pitting corrosion behavior of ultrafine-grained aluminum processed by ECAP: A statistical analysis. Mater. Corros..

[35] Ramgopal T., Gouma P.I., Frankel G.S. (2002). Role of Grain-Boundary Precipitates and Solute-Depleted Zone on the Intergranular Corrosion of Aluminum Alloy 7150. Corrosion.

[36] Xu D.K., Birbilis N., Lashansky D., Rometsch P.A., Muddle B.C. (2011). Effect of solution treatment on the corrosion behaviour of aluminium alloy AA7150: Optimisation for corrosion resistance. Corros. Sci..

[37] Lewandowska M., Wawer K., Kozikowski P., Ohnuma M., Kurzydlowski K.J. (2014). Precipitation in a nanograined 7475 aluminium alloy—Processing, properties and nanoanalysis. Adv. Eng. Mater..

[38] Chrominski W., Wenner S., Marioara C.D., Holmestad R., Lewandowska M. (2016). Strengthening mechanisms in ultrafine grained Al-Mg-Si alloy processed by hydrostatic extrusion—Influence of ageing temperature. Mater. Sci. Eng. A.

[39] Chrominski W., Lewandowska M. (2016). Precipitation phenomena in ultrafine grained Al-Mg-Si alloy with heterogeneous microstructure. Acta Mater..

[40] Deschamps A., De Geuser F., Horita Z., Lee S., Renou G. (2014). Precipitation kinetics in a severely plastically deformed 7075 aluminium alloy. Acta Mater..

[41] Gopala Krishna K., Sivaprasad K., Sankara Narayanan T.S.N., Hari Kumar K.C. (2012). Localized corrosion of an ultrafine grained Al-4Zn-2Mg alloy produced by cryorolling. Corros. Sci..

[42] Wawer K., Lewandowska M., Kurzydlowski K.J. (2012). Precipitate strengthening of nanostructured aluminium alloy. J. Nanosci. Nanotechnol..

[43] Wawer K., Lewandowska M., Kurzydlowski K.J. (2007). The influence of hydrostatic extrusion on size, shape, and spatial distribution of intermetallic particles in 7475 aluminium alloy. Mater. Eng..

[44] Ayer R., Koo J.Y., Steeds J.W., Park B.K. (1985). Microanalytical study of the heterogeneous phases in commercial Al-Zn-Mg-Cu alloys. Metall. Trans. A.

[45] Meng G., Wei L., Zhang T., Shao Y., Wang F., Dong C., Li X. (2009). Effect of microcrystallization on pitting corrosion of pure aluminium. Corros. Sci..

[46] Dobkowska A., Sotniczuk A., Bazarnik P., Mizera J., Garbacz H. (2021). Corrosion behavior of cold-formed aa5754 alloy sheets. Materials.

[47] Moore K.L., Sykes J.M., Hogg S.C., Grant P.S. (2008). Pitting corrosion of spray formed Al-Li-Mg alloys. Corros. Sci..

[48] Benedetti A., Cirisano F., Delucchi M., Faimali M., Ferrari M. (2016). Potentiodynamic study of Al-Mg alloy with superhydrophobic coating in photobiologically active/not active natural seawater. Colloids Surf. B Biointerfaces.

[49] Puiggali M., Zielinski A., Olive J.M., Renauld E., Desjardins D., Cid M. (1998). Effect of microstructure on stress corrosion cracking of an Al-Zn-Mg-Cu alloy. Corros. Sci..

[50] Knight S.P., Birbilis N., Muddle B.C., Trueman A.R., Lynch S.P. (2010). Correlations between intergranular stress corrosion cracking, grain-boundary microchemistry, and grain-boundary electrochemistry for Al–Zn–Mg–Cu alloys. Corros. Sci..

[51] Kairy S.K., Turk S., Birbilis N., Shekhter A. (2018). The role of microstructure and microchemistry on intergranular corrosion of aluminium alloy AA7085-T7452. Corros. Sci..

[52] Andreatta F., Terryn H., De Wit J.H.W. (2004). Corrosion behaviour of different tempers of AA7075 aluminium alloy. Electrochim. Acta.

[53] Chromiński W. (2016). Microstructural Heterogeneities and Their Influence on Precipitation Phenomena in a Severely Deformed 6082 Aluminium Alloy. Ph.D. Thesis.

